# Reducing Neonatal Mortality in Nepal’s Remote Regions: A Narrative Review of Challenges, Disparities, and the Role of Helping Babies Breathe (HBB)

**DOI:** 10.3390/pediatric17020048

**Published:** 2025-04-17

**Authors:** Victoria Jane Kain, Ranjan Dhungana, Animesh Dhungana

**Affiliations:** 1 School of Nursing and Midwifery, Brisbane South Campus, Griffith University, 170 Kessels Road, Brisbane, QLD 4111, Australia; 2 Safa Sunaulo Nepal, Kathmandu 44600, Nepal; ranjandh@gmail.com; 3 Choice Humanitarian, Kathmandu 44600, Nepal; animeshdh5@gmail.com; 4 Choice Humanitarian and Latter-Day Saint Charities’ (LDSC), Kathmandu 44600, Nepal

**Keywords:** neonatal mortality, Nepal, remote regions, rural healthcare, healthcare disparities, health equity, skilled birth attendants (SBAs), neonatal resuscitation, helping babies breathe (HBB), resource-limited settings, comprehensive emergency obstetric and neonatal care (CEmONC)

## Abstract

Background: Nepal’s diverse geography creates significant challenges for healthcare accessibility, particularly for neonatal care. Rural areas, especially in the mountainous regions, face severe healthcare gaps due to isolation, inadequate infrastructure, and a shortage of skilled staff. Strengthening healthcare in these underserved regions is essential to reducing neonatal mortality. Helping Babies Breathe (HBB) is a neonatal resuscitation training program designed to reduce neonatal mortality due to birth asphyxia in low-resource settings. Methods: A comprehensive literature search identified studies on neonatal mortality and interventions, particularly HBB, which were analyzed using a narrative synthesis approach. This review examines disparities in neonatal health outcomes, regional differences, and barriers to healthcare access. Findings: This review identifies key themes related to healthcare disparities, neonatal mortality, and birth outcomes in Nepal’s remote regions. Geographical isolation, inadequate healthcare infrastructure, and cultural barriers contribute to persistently high neonatal mortality, particularly in mountainous areas such as Jumla and Dolpa, where rates exceed 60 per 1000 live births. HBB has shown a significant impact, reducing neonatal mortality by up to 60% when effectively implemented. However, infrastructural gaps, lack of emergency transport, and the uneven distribution of skilled birth attendants (SBAs) remain critical challenges. Addressing these disparities requires expanded training, increased availability of neonatal resuscitation equipment, and culturally sensitive healthcare strategies.

## 1. Introduction

Nepal continues to face stark health disparities in neonatal outcomes, particularly in its remote and mountainous regions. Despite improvements in national indicators, many newborns in these areas remain at significantly higher risk of mortality due to persistent barriers in healthcare access, weak infrastructure, and entrenched cultural practices. This introduction outlines the multifaceted drivers of neonatal mortality in Nepal’s most underserved regions, focusing on three interconnected domains: geographical barriers, health system limitations, and persistent health inequities.

### 1.1. Geographical Barriers to Healthcare Access

Nepal’s topography presents major challenges to healthcare accessibility, particularly for neonatal and maternal care. The country is divided into three ecological zones: the Terai (plains), hilly regions, and mountainous regions. While the Terai and lower hills benefit from better connectivity and healthcare reach, mountainous districts—comprising 15% of the country’s landmass—experience chronic under-access due to rugged terrain, sparse settlements, and harsh climatic conditions [1,2].

Communities in districts such as Dolpa and Jumla often lack reliable road networks or functioning emergency transport systems, leading to delays in seeking and receiving care [3,4]. In many cases, travel to the nearest health facility can take several hours or even days on foot [5]. Air evacuation is rarely feasible due to cost and availability [6]. These factors create life-threatening delays during obstetric emergencies, contributing to neonatal mortality rates that exceed 60 per 1000 live births in some districts [7,8]. Addressing these barriers requires improvements in local healthcare infrastructure, in emergency transport systems, and in the availability of trained healthcare personnel in remote areas. A study in 2000 suggested that up to 36,443 neonatal deaths in Nepal could be prevented by 2030 if comprehensive interventions were implemented to enhance healthcare accessibility [8]. Comparable evidence from other low-resource countries, including Indonesia, highlights the life-saving impact of rapid access to institutional delivery care and skilled birth attendance in remote areas [9].

### 1.2. Healthcare Infrastructure and Access in Nepal

While geography exacerbates delays in care, health system limitations further restrict access to timely and quality neonatal services. Nepal’s healthcare system remains highly centralized, with most advanced medical facilities located in urban centers [10]. As of 2021, there were 3789 health posts and 3176 sub-health posts across the country, yet many rural and mountainous facilities operate with minimal staffing and critical supply gaps [11].

Although there is a tiered referral system, its functionality breaks down in remote districts such as Mugu and Humla, where hospitals are several hours away by foot and ambulances are often unavailable [12]. This leads many women to rely on traditional birth attendants—typically untrained—who are more accessible but ill-equipped to handle complications [13]. Cultural beliefs often discourage facility-based births, further perpetuating unsafe delivery practices [14].

Improving neonatal survival in these regions requires not only infrastructural investment but also human resource strengthening. Expanding the rural health workforce, ensuring a consistent supply of essential neonatal medicines and equipment, and building emergency referral pathways must be prioritized [2,6,15].

### 1.3. Neonatal Mortality and Health Disparities in Remote Nepal

While Nepal has made progress in reducing overall neonatal mortality, significant regional disparities remain. The national neonatal mortality rate (NMR) stands at 21 per 1000 live births yet, in remote mountainous regions, the rate surpasses 60 per 1000 [6,7]. Dolpa District, in particular, reports an NMR of 67 per 1000 live births, one of the highest in the country. The broader mountain region maintains an average neonatal mortality rate of 46 per 1000, nearly double the national average [13]. A major factor contributing to high neonatal mortality in remote Nepal is the prevalence of home births, often occurring in unsafe environments such as animal sheds due to traditional beliefs and customs [8]. In some regions, over 60% of neonatal deaths are linked to preventable causes such as hypothermia, infection, and birth asphyxia, conditions that could be managed effectively with basic neonatal care [14]. These statistics demonstrate the stark inequalities in neonatal health outcomes between urban and remote areas.

The lack of skilled healthcare providers further exacerbates these challenges, as many births occur without medical assistance [16]. Studies have shown that, while home births can be safe under optimal conditions, the lack of skilled birth attendants and emergency referrals significantly increases neonatal mortality risks in remote Nepal [17]. In contrast, some regions report poor hospital outcomes due to overcrowding, infection control issues, and lack of adequately trained neonatal care providers [18].

Reducing neonatal mortality due to birth asphyxia remains a pressing challenge in low-resource settings, particularly in Nepal’s remote regions where the absence of emergency transport and delays in seeking care mean that timely medical intervention is rarely available [19]. Addressing these disparities requires a multi-pronged strategy, including increasing the number of trained healthcare workers in rural districts [20] and implementing evidence-based neonatal resuscitation initiatives. One such program is Helping Babies Breathe (HBB), a globally recognized training curriculum specifically designed for resource-limited environments to improve newborn outcomes through timely resuscitation [21]. Expanding programs like HBB, alongside the development of culturally sensitive, community-based healthcare initiatives that promote safe birth practices, can help reduce preventable newborn deaths and narrow the health equity gap between urban and rural populations [22]. Without such targeted interventions, neonatal mortality in Nepal’s underserved areas will likely remain unacceptably high.

## 2. Methods

This review employs a narrative synthesis approach to analyze disparities in neonatal mortality and healthcare access in Nepal’s remote regions. Due to the heterogeneity of study designs, populations, and interventions, a quantitative meta-analysis was not feasible. Instead, Popay et al.’s Narrative Synthesis Framework [23] was utilized, as it provides a structured methodology to integrate findings from diverse studies and systematically identify thematic patterns across different contexts [24].

### 2.1. Search Strategy and Study Selection

A systematic literature search was conducted using electronic databases (PubMed, CINAHL, Embase, and Scopus) to identify relevant studies published within the last 25 years. Boolean operators (AND/OR) were applied to refine the search strategy, ensuring comprehensive coverage of key topics. The following search terms were used:

- ‘neonatal mortality’ OR ‘infant mortality’.
- ‘neonatal resuscitation’ OR ‘Helping Babies Breathe (HBB)’.
- ‘newborn care’ OR ‘birth outcomes’.
- ‘healthcare disparities’ OR ‘healthcare access’.
- ‘skilled birth attendants’ OR ‘healthcare workers’.
- ‘remote regions’ OR ‘rural healthcare’ OR ‘resource-limited settings’.
- ‘geographical barriers’ OR ‘terrain’ OR ‘infrastructure challenges’.
- ‘Nepal’ AND ‘hilly regions’ OR ‘mountainous regions’.
- ‘antenatal care’ OR ‘delivery practices’.
- ‘cultural barriers’ OR ‘socio-cultural factors’ OR ‘ethnic groups’.
- ‘healthcare interventions’ OR ‘healthcare programs’.

The search strategy was adapted to match each database’s syntax. No language restrictions were applied, although non-English studies were included only if English translations were available.

#### Inclusion and Exclusion Criteria

Studies eligible for this review focused on neonatal health in Nepal’s rural and remote regions, particularly in hilly and mountainous districts. Research on interventions such as HHB, antenatal care, birth practices, and SBAs was included if it explicitly reported neonatal mortality, birth outcomes, or healthcare disparities in these regions.

Various study designs were considered, including the following:

- Quantitative and qualitative studies;
- Randomized controlled trials;
- Observational studies.

Studies were excluded if they did not focus specifically on neonatal health in Nepal. However, research addressing maternal health was included if neonatal and maternal findings were reported separately. Studies that did not differentiate between hilly and mountainous areas were still considered if they focused on rural and remote healthcare challenges in Nepal.

### 2.2. Data Extraction and Analysis

A total of 334 papers were retrieved from across four major databases, with 62 from Scopus, 84 from Embase, 96 from PubMed, and 92 from CINAHL. After the removal of 248 duplicates, 86 unique studies remained for evaluation. The initial title and abstract screening led to the exclusion of 54 papers, leaving 32 studies for full-text review. Following a detailed assessment, 11 additional studies were excluded, resulting in a final sample of 21 relevant studies that met the inclusion criteria.

Data from the selected studies were extracted using a standardized data extraction form, capturing information such as the following:

- Study design and population;
- Neonatal mortality rates and birth outcomes;
- Healthcare access and service availability;
- Effectiveness of interventions;
- Geographical and cultural factors influencing neonatal healthcare.

**Figure 1:**
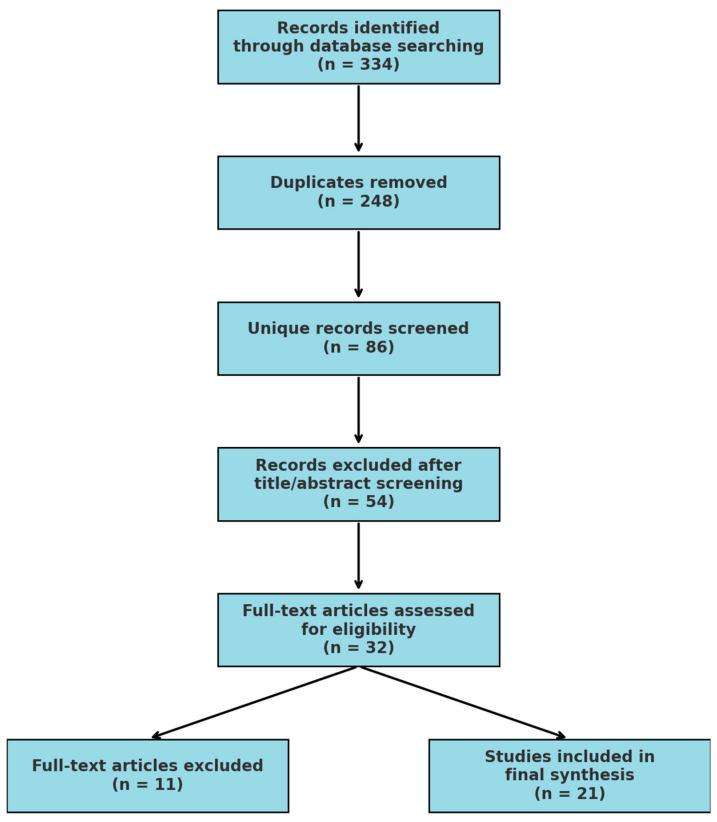
PRISMA flowchart for study selection.

A narrative thematic analysis was conducted to identify commonalities and variations in neonatal mortality trends, intervention effectiveness, and policy gaps contributing to persistent disparities. The PRISMA flowchart was used to guide the study selection process, ensuring transparency and methodological rigor (see Figure 1).

Findings were synthesized across both qualitative and quantitative studies, combining statistical trends with contextual healthcare challenges to provide a holistic understanding of neonatal healthcare disparities in Nepal’s remote settings.

### 2.3. Narrative Synthesis

Narrative synthesis is particularly relevant in healthcare and policy research, where interventions and contextual factors vary widely [25]. This approach facilitates an in-depth interpretation of complex health disparities and policy gaps by identifying relationships and trends in neonatal healthcare provision within Nepal’s rural and mountainous regions.

#### 2.3.1. The Four-Stage Narrative Synthesis Framework

Following Popay et al.’s framework [23], the synthesis was conducted in four distinct stages:

1. Preliminary synthesis;
2. Exploring relationships within and between studies;
3. Critical appraisal of studies;
4. Synthesis and presentation of findings.

#### 2.3.2. Preliminary Synthesis

A comprehensive literature search was conducted using PubMed, CINAHL, Embase, and Scopus to identify studies focusing on neonatal mortality and healthcare disparities in remote regions of Nepal. Articles were screened according to predefined eligibility criteria (detailed in in Section 2.1.: Inclusion and Exclusion Criteria), and key themes were identified using an inductive coding approach. A narrative summary of findings was compiled to highlight emerging patterns related to healthcare accessibility, effectiveness of interventions, and regional disparities in neonatal care.

#### 2.3.3. Exploring Relationships Within and Between Studies

To further analyze cross-study relationships, findings were categorized and stratified based on the following:

- Study type (qualitative vs. quantitative);
- Geographical region (hilly vs. mountainous);
- Type of intervention (e.g., HBB, SBA programs, etc.).

This thematic classification allowed for the examination of how geographical, infrastructural, and cultural disparities impact neonatal mortality. Differences in neonatal outcomes between rural and urban settings were also evaluated, considering healthcare infrastructure, socio-cultural influences, and study heterogeneity.

#### 2.3.4. Critical Appraisal of Studies

To ensure the credibility and reliability of the synthesis, all included studies underwent critical appraisal using a modified Critical Appraisal Skills Programme (CASP) checklist for qualitative and observational research [26]. Key areas assessed included the following:

- Study design and methodology;
- Sample size and representativeness;
- Intervention effectiveness and bias evaluation.

Particular attention was given to the risk of bias, including small sample sizes, self-reported data limitations, and potential publication bias favoring urban-based healthcare interventions.

### 2.4. Synthesis and Presentation of Findings

The final synthesis integrated key insights into four overarching themes:

1. Regional disparities in neonatal mortality and birth outcomes;
2. Impact of neonatal resuscitation programs such as HBB;
3. Infrastructure and resource gaps in neonatal healthcare;
4. Geographical barriers to healthcare access.

To enhance clarity, data were systematically tabulated, highlighting key differences in neonatal outcomes across Nepal’s rural districts. A conceptual model was also developed to illustrate the interconnections between these themes, offering a comprehensive synthesis of neonatal healthcare disparities and intervention effectiveness.

## 3. Results

The included studies represent a range of methodological approaches, including cross-sectional analyses, qualitative interviews, mixed-methods research, spatial assessments of healthcare accessibility, and community-based prospective cohort studies. These studies provide comprehensive insights into neonatal mortality rates, birth outcomes, healthcare disparities, and the effectiveness of interventions across different regions of Nepal (see Table 1). Key themes emerging from the synthesis included the following:

- Regional disparities in neonatal mortality and birth outcomes;
- The impact of neonatal resuscitation programs such as HBB, and infrastructure and equipment gaps in neonatal healthcare;
- Geographic barriers to healthcare access.

## 4. Discussion

This review synthesizes findings from the included studies examining neonatal mortality and healthcare disparities in remote regions of Nepal. This analysis highlights four key themes influencing neonatal health outcomes: regional disparities in neonatal mortality and birth outcomes, the impact of neonatal resuscitation programs such as HBB, infrastructure and resource gaps in neonatal healthcare, and geographical barriers to healthcare access. These themes are interlinked and reflect the compounded structural and systemic challenges facing neonatal health systems in Nepal’s most remote districts.

Significant regional disparities in neonatal mortality and birth outcomes persist across Nepal, particularly between remote mountainous areas and more accessible rural or urban regions. Bhattarai et al. [27] found that low birth weight prevalence was notably higher in hilly and mountain regions, with disparities influenced by maternal age, caste, and antenatal care access. Similarly, Karki and Kittel [7] reported persistently high neonatal mortality rates in Dolpa, where cultural resistance to institutional births and a reliance on untrained birth attendants continue to hinder neonatal survival.

One particularly hazardous practice in these areas is the occurrence of births in animal sheds, a custom that often involves no skilled attendance and takes place in highly unhygienic conditions. In 2000, Thapa et al. [8] documented that neonatal mortality was nearly three times higher in animal-shed births than in home births, largely due to unsanitary conditions, the absence of trained assistance, and heightened risks of infection and hypothermia.

Devkota and Bhatta (2011) [34] confirmed that this practice persists in some districts, with these births frequently unattended by SBAs, posing significant risks to both mothers and newborns. While other forms of home births may carry lower risks under trained supervision, animal-shed births typically occur without any medical oversight. The cultural practice of Chhaupadi, as explored by Joshi (2022) [35], reinforces these unsafe birthing conditions. Women are isolated in sheds during menstruation and childbirth, often due to traditional taboos. Despite government efforts to eliminate this harmful practice, it remains entrenched in some communities. Kaphle et al. [22] further noted that, in regions like Mugu, traditional beliefs often override medical advice, discouraging timely use of maternal health services. These insights underscore the pressing need for culturally grounded interventions that promote the presence of skilled birth attendants while addressing deep-rooted social norms.

Building trust through community engagement has proven effective in addressing such cultural barriers. For instance, the Community-Based Newborn Care Program (CB-NCP) in Nepal has shown promise by using Female Community Health Volunteers (FCHVs) to provide home education, facilitate referrals, and act as cultural intermediaries [36]. Similarly, CARE Nepal’s Safe Motherhood Projects employed peer-led women’s groups and local maternal health promoters to improve birth preparedness and shift harmful practices. Studies in similar South Asian contexts (e.g., Bangladesh and northern India) have shown that participatory learning action (PLA) groups—where women collectively reflect on and address childbirth norms—can significantly reduce neonatal mortality [37]. These examples reinforce the importance of co-created, community-driven strategies in driving lasting behavioral change.

However, structural challenges persist even when cultural barriers are addressed. The availability of SBAs varies significantly by region, impacting neonatal outcomes. Choulagai [17] found that only 48% of births in mid- and far-western Nepal were attended by SBAs, with distance from healthcare facilities and lack of transport cited as the main barriers. In Solukhumbu, Thomas et al. [32] reported that 35.7% of births occurred in health facilities, but many women still opted for home births due to limited transportation and facility shortages. Similarly, Schoenhals et al. [30] found that 70% of births in Solukhumbu were home deliveries, with many lacking skilled medical assistance.

Regional disparities extend to healthcare accessibility and neonatal survival rates. Kc et al. [28] found that neonatal mortality declined overall between 2001 and 2016, yet wealth-based disparities widened, with poorer families projected to meet neonatal survival targets much later than wealthier groups. These patterns signal that improving access alone is insufficient without targeted equity-focused policies.

In this context, the HBB program represents a critical intervention. Naresh et al. [21] reported a 60% reduction in neonatal mortality following proper implementation of HBB training. However, the program faces systemic limitations in rural areas. Tamang et al. [18] noted that high healthcare worker turnover and the absence of refresher training weakened its long-term efficacy.

Moreover, Pandey et al. [19] found that 91.6% of health facilities stocked neonatal resuscitation bags, yet skill retention among healthcare workers was poor, limiting the program’s overall effectiveness. Singh and Shankar [31] emphasized critical gaps in CPR knowledge, revealing that only 12.8% of Nepalese health assistants had received CPR training—and none had ever performed a live resuscitation. These gaps highlight a troubling disconnect between equipment availability and actual clinical capacity.

Ghimire et al. [6] further noted that, while cases of neonatal asphyxia declined in HBB-implemented centers, postnatal complications remained high due to limited referral options and poor follow-up care. Complications such as neonatal sepsis, hypothermia, and feeding difficulties require sustained, facility-based management that remains inaccessible in many rural regions. Maru et al. [29] recommend expanding in situ mentorship and supportive supervision to improve training outcomes and reinforce HBB effectiveness.

Thus, scaling the HBB program must go hand-in-hand with broader systemic reforms—including frequent refresher training, incentives for rural postings, and stronger neonatal referral networks. Without this support, the impact of HBB will remain uneven and constrained.

Severe infrastructure and resource shortages continue to undermine neonatal survival in Nepal’s remote regions. Bhattarai et al. [27] found that many health facilities lacked essential neonatal care infrastructure, including electricity, incubators, and sterile equipment. Similarly, Tuladhar [33] reported that, by 2021, only 2.2% of health facilities stocked all essential neonatal medicines, demonstrating a major gap in facility readiness for neonatal emergencies.

Facilities in high-mortality districts, such as Dolpa and Jumla, are particularly ill-equipped for neonatal care. Karki and Kittel [7] noted frequent medicine shortages, while Tamang et al. [18] found that most facilities lacked functional incubators and transport services. Thomas et al. [32] further highlighted staff shortages, with no trained obstetric and neonatal emergency personnel available at many rural health posts.

Comprehensive Emergency Obstetric and Neonatal Care (CEmONC) standards require healthcare facilities to be equipped to handle emergency maternal and neonatal complications, including cesarean sections and neonatal resuscitation. Acharya et al. [20] found that only 16.1% of Nepalese health facilities were equipped to manage assisted vaginal deliveries. Expanding these services could help address gaps not just in emergency care but also in routine neonatal health delivery. Maru et al. [29] demonstrated that investing in CEmONC centers boosted institutional births and reduced neonatal mortality in underserved districts.

Integrating neonatal interventions such as HBB within these centers enhances reach and impact. Målqvist et al. [38] observed a 36% reduction in early neonatal mortality when HBB was combined with CEmONC support. Kc et al. [28] documented declines in birth asphyxia post-HBB training, and Shrestha et al. [13] emphasized that outcomes improve substantially when HBB is embedded within a functioning referral and emergency care system. These findings reinforce the importance of a systems-based approach—linking community-level resuscitation to facility-based emergency care.

Geographical isolation compounds all these challenges. Paudel et al. [16] reported that, in remote Jumla villages, women often walked 7–8 h to reach the nearest facility. In Dolpa, poor roads and rugged terrain similarly hinder timely access to maternal care [7]. Tamang et al. [18] found that 20 out of 31 health facilities in Jumla lacked ambulances, severely limiting referral services. Cao et al. [2] showed that access to motorized transport significantly reduced neonatal mortality—but most mountainous districts lacked even basic infrastructure. These logistical constraints often turn treatable conditions into fatal outcomes.

Solutions must include the expansion of community-based birthing centers and improved transportation networks. Shrestha and Jung [13] recommend mobile outreach and rural ambulance programs to close the accessibility gap and ensure continuity of care for neonates post-delivery.

## 5. Recommendations

Expanding access to SBAs should be the overarching recommendation for reducing neonatal mortality in Nepal’s remote regions. SBAs form the foundation of safe neonatal care and can directly facilitate the implementation of other interventions such as HBB, telemedicine, and emergency referral systems. Without a trained birth workforce in place, even the best-designed innovations struggle to produce long-term impact.

Future research should prioritize the development of sustainable SBA-based neonatal resuscitation training models, tailored to the challenges faced by healthcare workers in remote Nepal. While programs such as HBB have demonstrated success, studies indicate that skill retention declines over time without reinforcement, especially in settings where access to refresher training is limited [21,31].

The role of digital and remote learning tools in SBA-centered neonatal training requires further investigation, particularly in regions with limited internet and electricity access. Research should evaluate offline-accessible training models, as well as strategies to support healthcare worker engagement and knowledge retention through blended or hybrid learning. SBA presence is also essential for the effective use of telemedicine and mobile health (mHealth). Future studies should explore how real-time neonatal case support and remote consultations influence SBA decision-making and outcomes in rural facilities [2,19,28].

The impact of supply chain inefficiencies on SBA performance in high-mortality districts should also be evaluated. While previous studies document shortages of essential medicines and neonatal equipment [27,33], research should now focus on intervention models that support SBA-led care through improved logistics and facility readiness.

Alternative emergency referral systems should be studied in relation to SBA deployment. Rural ambulance networks, air transport, and mobile maternal health units could enhance SBA ability to respond to critical neonatal emergencies [16,18]. Finally, future studies should assess how provincial and national policies influence SBA recruitment, training, and rural retention, with an emphasis on identifying policy reforms that strengthen long-term maternal and neonatal care delivery [13].

## 6. Limitations

This review is limited by the heterogeneity of the included studies and potential selection bias, as a quantitative meta-analysis was not feasible. Additionally, reliance on published literature may exclude unpublished but relevant community-level interventions.

## 7. Conclusions

The findings from this review highlight four major barriers to neonatal healthcare in Nepal’s remote regions. Stark regional disparities persist in neonatal survival rates, exacerbated by socioeconomic inequities and cultural birth practices. While neonatal resuscitation programs such as HBB have demonstrated effectiveness, training gaps and healthcare worker turnover reduce their long-term impact. Infrastructure and resource limitations severely restrict neonatal care capacity, while geographic barriers continue to prevent timely healthcare access.

Addressing these systemic challenges requires comprehensive, multi-level interventions, including expanded SBA training, enhanced emergency transport systems, and investments in healthcare infrastructure. While existing programs such as HBB and CEmONC have shown promise, their success depends on addressing underlying systemic inequities in Nepal’s most underserved regions.

## Figures and Tables

**Table 1:** Summary of included papers.

Author Year	Study Design	Study Population	Key Themes	Findings	CASP Score [26]
Acharya et al. (2021) [20]	Cross-sectional	457 health facilities across Nepal	Healthcare infrastructure, facility readiness, emergency obstetric care	Hospitals had higher neonatal care readiness. Only 16.1% offered assisted vaginal birth; 10% provided anticonvulsants. Readiness was linked to staffing levels, 24 h service, and newborn death review.	Moderate risk of bias due to self-reported facility readiness assessments
Bhattarai et al. (2022) [27]	Cross-sectional.	1386 term singleton births from 4 hospitals in eastern Nepal	Geographic disparities, birth weight, maternal factors	Low birth weight was more prevalent in hilly regions (6.6%). Dalit ethnicity, low maternal age, and higher antenatal visits associated with hilly-region births.	Low risk; large dataset but limited causal inferences
Cao et al. (2021) [2]	Spatial analysis	5553 public health facilities, 2020 population data	Healthcare access, geographic barriers	A total of 92.54% of the population accessed facilities within 15 min via motorized transport. Accessibility declined for higher-level facilities. Recommended new health centers in underserved areas.	Moderate risk due to reliance on modeled transport data
Choulagai (2013) [17]	Cross-sectional	2481 women who gave birth in last 12 months in three districts	Skilled birth attendants, barriers to healthcare access	Barriers included distance (45%) and transport issues (21%). A total of 48% used SBAs. Antenatal care improved SBA utilization.	Low risk; strong sample representation but lacks qualitative depth
Ghimire (2019) [6]	Demographic health survey analysis	23,335 pregnancies from Nepal Demographic Health Survey	Neonatal mortality trends, regional disparities	Perinatal mortality rate was 42 per 1000 births. Higher mortality in mountainous regions, younger mothers, poor sanitation.	Low risk; robust dataset but lacks intervention-specific data
Kaphle et al. (2013) [22]	Qualitative	25 pregnant/postnatal women, 16 healthcare/community stakeholders in Mugu	Cultural barriers, birth practices	Animal-shed births preferred due to spiritual beliefs, leading to neonatal risks. Cultural beliefs conflicted with medical advice.	Moderate risk due to small sample size and subjective reporting
Karki and Kittel (2019) [7]	Mixed-methods	12,287 people from Dolpa District	Neonatal mortality, cultural influences, healthcare access	Neonatal mortality rate was 67 per 1000. Cultural mistrust of modern medicine and poor health infrastructure led to increased deaths.	Moderate risk; strong sample but relies on retrospective data
Kc et al. (2017) [28]	Secondary analysis	Women aged 15–49 from Nepal Demographic and Health Surveys (2001, 2006, 2011, 2016)	Neonatal mortality trends, socioeconomic disparities	Neonatal mortality decreased between 2001 and 2016, but disparities widened between wealth quintiles. Tetanus vaccination status, maternal education, and household conditions were key predictors of neonatal mortality.	Low risk; robust dataset but limited ability to analyze causal relationships
Khanal et al. (2024) [15]	Prospective cohort	735 mother–infant pairs in western Nepal	Home births, healthcare utilization	Total of 11.8% had home births. Low antenatal care increased likelihood of home birth. Higher wealth correlated with hospital births.	Low risk; strong methodology but lacks long-term neonatal tracking
Khatri et al. (2022) [14]	Cross-sectional	901 antenatal care facilities and 454 perinatal service providers	Healthcare infrastructure, service quality	Structural quality scores were higher for private facilities; government-run facilities in rural areas showed poor readiness for maternal and newborn care.	Moderate risk; self-reported facility assessments limit objectivity
Maru et al. (2017) [29]	Pre-post intervention	210 postpartum women in rural Nepal	Emergency obstetric care, birth facility utilization	Institutional birth rates rose from 30% to 77% after CEmONC introduction. Availability improved birth planning and safety perceptions.	Low risk; rigorous comparison but limited to one hospital area
Naresh et al. (2022) [21]	Prospective observational	18 health facilities assessing 49,809 births	Newborn resuscitation, HBB	HBB training reduced neonatal deaths and birth asphyxia. Skill retention remained high over 24 months.	Low risk; large dataset, but results may not generalize to non-participating regions
Pandey et al. (2023) [19]	Cross-sectional	804 health facilities in Nepal	Emergency obstetric care, facility readiness	Service availability for neonatal care remains inadequate. Only 43.7% of facilities met Comprehensive Emergency Obstetric and Neonatal Care (CEmONC) standards.	Moderate risk due to facility-reported data
Paudel et al. (2018) [16]	Qualitative	42 interviews with women who experienced perinatal deaths and 20 interviews with healthcare workers	Healthcare system barriers, perinatal mortality	Poor governance, lack of community engagement, and weak health system accountability contribute to high perinatal mortality in remote villages.	Moderate risk; small sample limits generalizability
Schoenhals et al. (2017) [30]	Cross-sectional	122 women who gave birth in the past 24 months in Solukhumbu District	Maternal–newborn health practices	Only 26% of births occurred in health facilities, with 70% at home. Limited access to skilled birth attendants contributed to neonatal complications.	Moderate risk; small sample size but relevant to high-altitude populations
Shrestha and Jung (2023) [13]	Quasi-experimental	Rural Nepalese children from Nepal Living Standards Survey data	Healthcare reform, gender-based disparities	Healthcare reform reduced infant mortality for boys but had no significant effect on girls, suggesting persistent societal gender biases in healthcare access.	Moderate risk; reliance on secondary data may not capture all confounders
Singh and Shankar (2023) [31]	Cross-sectional	500 health assistants registered with Nepal Health Professional Council	CPR knowledge among healthcare workers	Only 12.8% had CPR training; none had performed CPR. Training gaps highlight urgent need for competency development.	Low risk; strong methodology but limited to one profession
Tamang et al. (2021) [18]	Descriptive cross-sectional	31 state-run health facilities in Jumla District	Facility preparedness, medicine availability	Many facilities lacked essential neonatal medicines and transport services. Emergency preparedness was inadequate in most centers.	Moderate risk; self-reported data may limit reliability
Thapa et al. (2000) [8]	Community-based retrospective	3007 live-born children from 772 mothers in Jumla, Nepal	Animal-shed births, neonatal mortality	Neonatal mortality was significantly higher for births occurring in animal sheds compared to homes. Lack of hygiene and medical care contributed to higher risk.	Moderate risk; older dataset but relevant to cultural birth practices
Thomas et al. (2022) [32]	Cross-sectional	487 households and 19 health facilities in Solukhumbu District	Facility readiness, SBA availability	Only 35.7% of births occurred in health facilities. Lack of trained obstetric and neonatal staff hindered service readiness.	Low risk; large sample, but self-reported barriers may introduce bias
Tuladhar (2024) [33]	Cross-sectional	Survey data from 2015 and 2021 assessing facility readiness	Neonatal service provision, healthcare trends	By 2021, only 2.2% of facilities stocked all essential neonatal medicines. Readiness for neonatal care remained critically low, particularly in rural areas.	Moderate risk; government survey data may not reflect all local disparities
